# Repeat robotic nephron-sparing surgery for metachronous multifocal tumors in a solitary kidney: a case report

**DOI:** 10.25122/jml-2025-0059

**Published:** 2025-04

**Authors:** Stelian Ianiotescu, Constantin Gingu, Irina Balescu, Nicolae Bacalbasa, Ioanel Sinescu

**Affiliations:** 1 Carol Davila University of Medicine and Pharmacy, Bucharest, Romania; 2 Center of Uronephrology and Kidney Transplantation, Fundeni Clinical Institute, Bucharest, Romania; 3 Department of Nephrology, Urology, Immunology and Immunology of Transplant, Dermatology, Allergology, Faculty of Medicine, Carol Davila University of Medicine and Pharmacy, Bucharest, Romania; 4 Department of Surgery, Carol Davila University of Medicine and Pharmacy, Bucharest, Romania; 5 Department of Visceral Surgery, Center of Excellence in Translational Medicine, Fundeni Clinical Institute, Bucharest, Romania; 6 Department of Visceral Surgery, Center of Digestive Diseases and Liver Transplantation, Fundeni Clinical Institute, Bucharest, Romania

**Keywords:** nephron-sparing surgery, metachronous multifocal tumor, solitary kidney, robotic surgery

## Abstract

We report the case of a 58-year-old male with metachronous renal tumors and a solitary kidney who had previously undergone an open right radical nephrectomy with extended lymphadenectomy for an invasive renal cell carcinoma (RCC) (pT3a N0M0) in November 2013. In May 2022, during routine surveillance, a left lower pole lesion measuring 2.5 × 2 × 1.6 cm was detected, and the patient was submitted to robot-assisted partial nephrectomy (RAPN). The histopathological study confirmed the presence of a pT1a Fuhrman grade 3 clear cell renal carcinoma. In October 2024, follow-up imaging revealed a new upper pole lesion measuring 4 × 3 × 2.3 cm in the left kidney. The patient was submitted to a novel robot-assisted partial nephrectomy, which was successfully completed using selective clamping of the renal artery. The clamping time was 28 minutes (versus 17 minutes during the initial procedure), and the estimated blood loss increased to approximately 300 mL compared to about 100 mL previously, with a console time of 98 minutes. The patient was discharged after the second surgery in good functional status. The final pathology revealed clear cell RCC, Fuhrman grade 2/nucleolar grade 2 (WHO/ISUP 2016), and pT1a, with negative margins. Despite increased technical challenges during reoperation, postoperative renal function remained stable, underscoring the feasibility of repeat RAPN in a solitary kidney.

## INTRODUCTION

Robot-assisted partial nephrectomy (RAPN) is a well-established, minimally invasive approach for nephron-sparing surgery (NSS) in patients with renal masses [1]. The preservation of renal parenchyma is crucial, especially in patients with a solitary kidney, to prevent chronic kidney disease and delay the need for renal replacement therapy [2]. Multifocal renal tumors—often seen in hereditary or syndromic conditions—complicate surgical planning, necessitating repeat resections while conserving as much functional renal tissue as possible [3,4].

## CASE PRESENTATION

**Figure 1:**
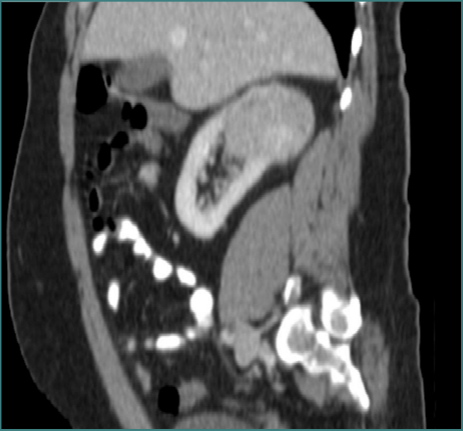
Large renal tumor at the level of the right kidney – an open radical right nephrectomy was performed at that moment

A 58-year-old male patient with metachronous renal tumors and a solitary kidney had previously undergone an open right radical nephrectomy with extended lymphadenectomy in November 2013 for an invasive RCC (pT3a N0M0) (Figure 1).

**Figure 2:**
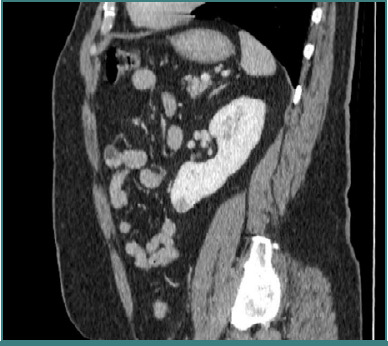
Computed tomography image of a small renal tumor at the level of the lower pole of the left kidney – a robotic partial nephrectomy was performed

In May 2022, routine surveillance revealed a 2.5 × 2 × 1.6 cm lesion in the lower pole of the left kidney (Figure 2). The patient subsequently underwent RAPN with negative margins. He was discharged with a serum creatinine of 1.24 mg/dL and an eGFR of 69.22 mL/min/1.73 m^2^. Histopathology confirmed clear cell RCC, Fuhrman grade 3/nucleolar grade 3 (WHO/ISUP 2016), staged as pT1a.

**Figure 3:**
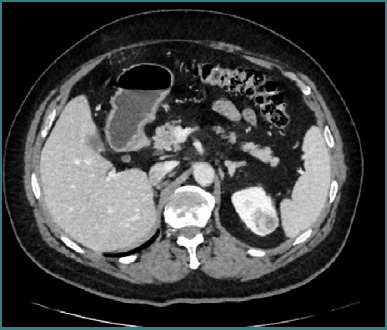
Follow-up computed tomography revealed the presence of another tumor at the level of the left upper renal pole

In October 2024, follow-up contrast-enhanced imaging detected a new 4 × 3 × 2.3 cm lesion in the upper pole of the left kidney (Figure 3).

**Figure 4:**
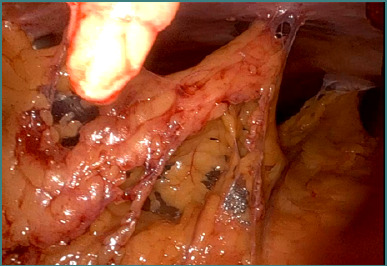
Initial intraoperative aspect – postoperative adhesions following partial nephrectomy in a patient with solitary kidney

**Figure 5:**
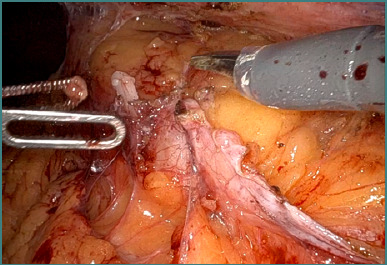
The final aspect after adhesiolysis – identification of the left kidney

**Figure 6:**
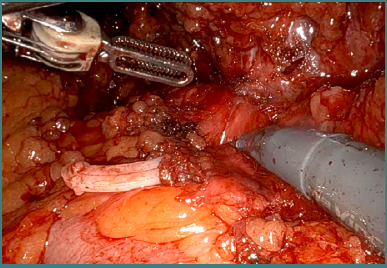
Intraoperative aspect – identification and dissection of the renal vascular pedicle

**Figure 7:**
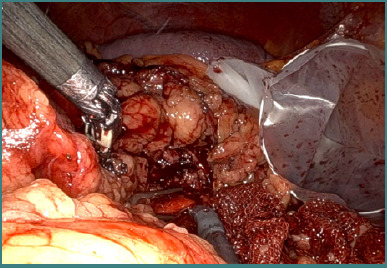
Selective clamping of the renal artery via the posterior approach

**Figure 8:**
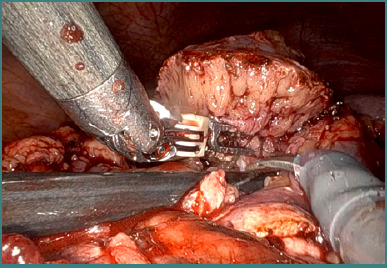
Enucleation of the tumoral mass

**Figure 9:**
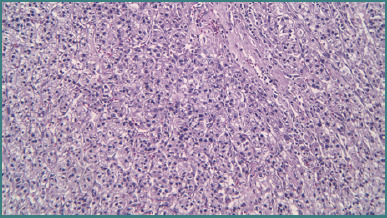
Hematoxylin-eosin (20X) demonstrating the presence of a renal cell carcinoma

Recognizing the challenges of multifocality—which may be associated with an underlying hereditary syndrome—the multidisciplinary team opted for a repeat RAPN on the solitary kidney. Intraoperative findings included dense adhesions (Figures 4 and 5) and significant perihilar fibrosis (Figures 6 and 7), complicating robotic docking and hilar dissection. Although standard practice recommends en bloc clamping of the renal artery and vein, only selective clamping of the renal artery was achievable after meticulous dissection of the posterior renal plane (Figures 6-8). The repeat procedure required a clamping time of 28 minutes (versus 17 minutes during the initial RAPN) and resulted in an estimated blood loss of approximately 300 mL compared to about 100 mL previously, with a console time of 98 minutes. Frozen section analysis confirmed negative margins and final pathology revealed clear cell RCC, Fuhrman grade 2/nucleolar grade 2 (WHO/ISUP 2016), pT1a (Figure 9). The patient was discharged after the second operation with a serum creatinine of 1.34 mg/dL and an eGFR of 51.71 mL/min/1.73 m^2^. Follow-up imaging at 3 and 12 months postoperatively showed no evidence of tumor recurrence.

## DISCUSSION

The detection of multifocal renal tumors can raise suspicion of an underlying hereditary or syndromic condition, even in the absence of a confirmed genetic diagnosis [3]. Multifocality necessitates a tailored surgical approach, often involving multiple resections (either enucleations or partial nephrectomies), with the primary goal of preserving as much renal parenchyma as possible [4-8]. This is critical for maintaining renal function because patients with multifocal disease have a higher risk of subsequent recurrences, requiring close and ongoing surveillance [2,9].

In such cases, repeated partial nephrectomy seems to be the key to achieving a good oncological and functional outcome. However, repeated RAPN in a solitary kidney presents unique technical challenges. Prior surgeries result in perinephric fibrosis and adhesions that obscure normal tissue planes [5,10]. In our case, extensive adhesiolysis was necessary to access the kidney, and careful dissection of the renal hilum was essential due to fibrosis surrounding the vascular structures. En bloc clamping of the renal hilum with a Satinsky clamp was employed to mitigate the risks associated with individual vessel dissection [1].

Given the possibility of multiple lesions in a multifocal setting, surgical planning must consider the potential need for future resections [11]. Therefore, every effort is made to preserve maximum renal parenchyma during tumor excision [4,6,8]. This may involve enucleoresection techniques that focus on removing only the tumor tissue while sparing the surrounding normal kidney [4]. However, the complex anatomy following previous surgeries may prolong operative time and warm ischemia time, which are known to impact postoperative renal function [2,12,13].

When it comes to the recurrence risk and follow-up of such cases, attention should be focused on the idea that patients with multifocal RCC are at a higher risk for local recurrence due to the underlying field defect associated with hereditary syndromes [14,15]. As a result, meticulous postoperative surveillance is essential to detect new lesions early and allow for timely intervention. Regular imaging, including contrast-enhanced MRI and functional renal assessments, remains the cornerstone of postoperative follow-up in these patients [5,16,17].

In this respect, we should not omit the fact that the complexity of preoperative renal surgery necessitates a multidisciplinary team approach involving urologists, radiologists, and anesthesiologists. Detailed preoperative planning, including a review of prior surgical records and imaging studies, is crucial for anticipating challenges and optimizing surgical strategy [18-20]. In this case, our team’s expertise allowed us to achieve oncologic control and preservation of renal function despite the technical difficulties posed by previous surgeries.

## CONCLUSION

This case of a 56-year-old male patient with a solitary kidney illustrates that repeat RAPN can be safely and effectively performed even in the presence of multifocal renal tumors—which may suggest a hereditary or syndromic component—and significant post-surgical adhesions. The technical challenges of dense fibrosis, complex dissection of the renal hilum, and the need for multiple resections were successfully managed by meticulous surgical techniques. Preservation of renal parenchyma and close postoperative follow-up are paramount in managing patients with multifocal disease due to the higher risk of recurrence. Our experience supports the role of repeat RAPN as a viable option to achieve oncologic and functional success in this challenging patient population.
